# Effects of Different Drying Methods on the Drying Characteristics and Quality of *Codonopsis pilosulae* Slices

**DOI:** 10.3390/foods12061323

**Published:** 2023-03-20

**Authors:** Yuanman Yue, Qian Zhang, Fangxin Wan, Guojun Ma, Zepeng Zang, Yanrui Xu, Chunhui Jiang, Xiaopeng Huang

**Affiliations:** College of Mechanical and Electronical Engineering, Gansu Agricultural University, Lanzhou 730070, China

**Keywords:** *Codonopsis pilosula*, drying methods, rotary microwave vacuum drying, quality, microstructure

## Abstract

The present study aimed to investigate the effect of rotary microwave vacuum drying (RMVD), radio frequency vacuum drying (RFVD), vacuum far infrared drying (VFID), vacuum drying (VD), hot air drying (HD) and natural drying (ND) on the drying characteristics, active ingredients and microstructure of *Codonopsis pilosulae* slices. Compared with the fitting results of the four models, the Weibull model is the most suitable drying model for *Codonopsis*. The RFVD and HD color difference values were smaller compared to ND. The effective moisture diffusivity (Deff) under different drying methods was between 0.06 × 10^−8^ m^2^/s and 3.95 × 10^−8^ m^2^/s. RMVD-dried products had the shortest drying time and retained more active ingredients. The microstructure analysis revealed that the porous structure of RMVD is more favorable for water migration. RMVD is a promising dehydration method for obtaining high-value-added dried Codonopsis products.

## 1. Introduction

Radix *Codonopsis* is the dried root of *Codonopsis pilosula* (Franch.) Nannf, *Codonopsis pilosula* Nannf. var. modesta (Nannf.), L.T. Shen or *Codonopsis tangshen* Oliv [1]. As a common medicine in traditional Chinese medicine prescriptions, *Codonopsis* has been certified to have the effects of tonifying middle qi, relieving cough, invigorating the spleen and benefiting the lungs, nourishing the blood and generating fluid [2,3]. *Codonopsis pilosula* polysaccharide, like other medicinal plant polysaccharides, has a wide range of pharmacological effects, including regulating body immunity, scavenging free radicals and hematopoiesis. Among these effects, the immune regulation effect of *Codonopsis pilosula* polysaccharide, which has been studied more frequently and deeply, is mainly manifested as an ability to enhance immune function, consistent with the effect of *Codonopsis pilosula* on tonifying qi, invigorating the spleen and benefiting the lungs [4]. Fresh *Codonopsis* usually contains 80–85% water, which is susceptible to microbial and chemical deterioration. Drying is the most commonly used process to inhibit microbial growth and delay the deterioration of biochemical reactions. It is also the basic processing method for obtaining by-products. However, the drying process may cause thermal damage and severe changes in the physical, chemical and organoleptic properties of the aromatic plant. Therefore, the choice of drying method is very important [5,6].

Hot air (HD) is considered to be a low-cost and easily controlled drying method. However, it is associated with the degradation of bioactive compounds, loss of color and changes in food structure [7,8], usually leading to the degradation of important flavor, color and nutritional compounds [9]. Far infrared drying (FIRD) is considered as a promising drying method for food products. When infrared radiation is used to dry food products of a high moisture content, the energy penetrates the materials to a small depth and is then converted into heat [10]. Compared with the traditional heating method, radio frequency vacuum drying (RFVD) has a volume heating effect which can significantly reduce the quality damage caused by uneven heating inside and outside the material during the traditional drying process [11]. Vacuum drying (VD) allows the material to be dried at a lower pressure, which avoids the oxidation of the sample and reduces the drying temperature, thereby preserving the appearance and quality of the dried product [12]. Nevertheless, it also has problems, such as a high energy consumption, high capital cost and a long drying time [13]. Microwave vacuum drying (MVD) is based on microwave radiation to transfer energy and heat the medium as a whole. The drying time is short, and the vacuum environment reduces the drying temperature of the material, avoiding a long drying time and reducing the energy consumption. In recent years, MVD has been widely used in the field of food drying [14]. In the MVD process, the temperature of the sample center is higher. Therefore, the water evaporation rate of the central part is faster than that of other parts, resulting in a lower water content in the central part of the dried product. This has been confirmed in the processing of agricultural products such as banana [15], ginger [16] and mango [17]. At present, the microwave vacuum drying equipment is mostly of a rotary-table type, and the distance between the material and the microwave source remains unchanged during the drying process. During continuous drying, due to the large drying intensity, the problem of uneven drying occurs easily. In severe cases, local overheating will lead to a burnt paste, affecting the quality of the dried products. Rotary microwave vacuum drying (RMVD) is different from the rotary-table type. The rotating wheel containing the material plate alternately approaches the microwave source by vertical rotation. While making full use of the advantages of microwave vacuum drying, it can effectively avoid local overheating, thus improving the drying uniformity of the material.

Unfortunately, there is no comparative analysis of the effects of different drying methods on the drying energy consumption, active ingredients, antioxidant capacity and microstructure of *Codonopsis pilosula*. The purpose of this study was to explore the feasibility of RMVD in *Codonopsis pilosula* processing. Therefore, the effects of different drying methods such as RMVD, RFVD, VFID, VD, HD and natural drying (ND) on the drying characteristics and quality of *Codonopsis pilosula* were compared.

## 2. Materials and methods

### 2.1. Raw Materials

The fresh *Codonopsis pilosula* used in this experiment was procured from Min County Guiqi Ginseng Growers Cooperative (Lanzhou, China). In order to prevent water loss of the fresh *Codonopsis*, it was purchased and placed in a 4 °C constant-temperature fresh-keeping cabinet. According to the official AOAC method, the average water content of fresh *Codonopsis pilosula* at 105 °C for 24 h was 73.5 ± 2% (w.b.).

### 2.2. Different Drying Processes

In this study, five different drying methods were used to study the drying characteristics and quality of *Codonopsis pilosula*. Before drying, the dirt and sand were washed off from the rhizomes. Filter paper was used to absorb excess water, and the rhizomes were cut into 4 ± 0.5 mm thick discs. Each drying method used a weighed 120 ± 0.5 g sample. All *Codonopsis* samples used in the experiment came from the same batch.

#### 2.2.1. Rotary Microwave Vacuum Drying (RMVD)

The RMVD equipment (HWZ-30) used in this experiment was jointly developed by the School of Mechanical and Electrical Engineering of Gansu Agricultural University (Lanzhou, China) and Tianshui Shenghua Microwave Vacuum Co., Ltd. (Tianshui, China) (Figure S1). The control system in the equipment can adjust the rotation speed, drying temperature and vacuum. Three independent microwave generators work directly above the drying chamber. The microwave wavelength is 122 mm, and the frequency is 2450 MHz. Automatically controlled by the PLC program, the input maximum power is 3 KW, and the effective microwave absorption rate is 1 kg.kw/h. The control system uses an infrared temperature sensor to monitor the temperature of the material, which can continuously detect the surface temperature. Meanwhile, the parameters can be recorded in the log file under the control of PLC. The slices of *Codonopsis pilosula* were weighed and put into the drying equipment cavity for the drying test. According to the preliminary test, the process parameters of the test were determined to be a 55 °C drying temperature and a 25 Hz rotation speed. The absolute pressure inside the chamber was set at 30 KPa. The samples were weighed every 3 min, and the changes in the sample weight and water content were recorded. When the water content dropped below the safe water content (10%) [1], the test was ceased. When weighing, the materials are taken out and weighed.

#### 2.2.2. Radio Frequency Vacuum Drying (RFVD)

The equipment used for the test was a GJS-3-27-JY high-frequency vacuum medium heating test device (Hebei Huashi Jiyuan High Frequency Equipment Co., Ltd., Langfang, China). The samples were weighed and laid on the material tray. According to the previous test, the samples were dried under the conditions of a plate spacing of 90 mm, a vacuum of 0.025 Mpa and a drying temperature of 55 °C. The samples were weighed every 20 min until the moisture content of the samples decreased to a safe moisture content (10%). The samples were removed and sealed after cooling. When weighing, the materials were taken out and weighed.

#### 2.2.3. Vacuum Drying (VD)

DZF-type vacuum drying equipment (Shanghai Lichen Bangxi Instrument Technology Co., Ltd., Shanghai, China) was used for the VD test. The absolute pressure inside the chamber was set at 30 KPa, and the drying temperature was 55 °C. The sample quality was weighed every 30 min in the early stage, and samples were weighed every 60 min in the later stage. The experiment was ceased when the moisture content of the sample was reduced to a safe moisture content (10%), and it was removed and sealed after cooling.

#### 2.2.4. Vacuum Far Infrared Drying (VFID)

The VFID test equipment was jointly developed by the School of Mechanical and Electrical Engineering of Gansu Agricultural University (Lanzhou, China) and Tianshui Shenghua Microwave Vacuum Co., Ltd. (Tianshui, China). The equipment consists of two parts: a heating system and a control system. A structural diagram is shown in Figure S2 [18]. The absolute pressure inside the chamber was set at 80 KPa. Before the test, the temperature was set at 55 °C. The sample was then placed on a sieve plate and placed in the oven for testing. The sample was weighed every 20 min until the water content of the sample was reduced to 10%. The sample was then removed, cooled, and sealed.

#### 2.2.5. Hot Air Drying (HD)

The HD process used a WGLL-230BE electric blast drying oven (Tianjin Taisite Instrument Co., Ltd., Tianjin, China). The air humidity was changed during the drying process. The air humidity was relatively large when drying commenced. As the drying process proceeded, the air humidity lessened. There were moisture discharge holes above the drying equipment. Unfortunately, the existing conditions in the laboratory were not certain for determining the air humidity. The cut *Codonopsis* slices were placed in a wire mesh plate, and the drying temperature was set to 55 °C. The samples were taken out every 20 min and weighed until the moisture content of the samples decreased to a safe moisture content (10%).

#### 2.2.6. Natural Drying (ND)

An empty space that was exposed to sunlight was selected. The stacked objects were laid and flattened in the empty space so that the stacked objects were flat. Next, a mesh cover with holes was expanded and laid on the stacked objects. The Codonopsis slices were stacked in the mesh cover and then flattened for drying.

#### 2.2.7. The Process

The process involved in this experiment is shown in Figure S3.

### 2.3. Analysis of Drying Characteristics

#### 2.3.1. Dry Base Moisture Content Determination

(1) $$M_{t}=\frac{m_{t}-m_{d}}{m_{d}}$$

where *m_t_* is the mass of *Codonopsis* at time *t* (g); *m_g_* is the dry matter mass of *Codonopsis* (g).

#### 2.3.2. Determination of Drying Rate

(2) $$DR=\frac{M_{t_{2}}-M_{t_{1}}}{t_{2}-t_{1}}$$

where *t*_2_ − *t*_1,_ *M_t_*_1_ and *M_t_*_2_ are the time difference and moisture content (d.b.) at times *t*_1_ and *t*_2_, respectively.

#### 2.3.3. Determination of Moisture Ratio

(3) $$MR=\frac{M_{t}-M_{e}}{M_{c}-M_{e}}$$

where *M_t_*, *M_c_* and *M_e_* are the time *t*; initial and equilibrium moisture content on a dry basis (d.b.), respectively.

Since the equilibrium water content of the *Codonopsis* in the experiments was relatively small (0.08–0.1 g water/g dry solid), Equation (3) can be simplified as Equation (4) [19]:

(4) $$MR=\frac{M_{t}}{M_{c}}$$

#### 2.3.4. Effective Moisture Diffusivity

(5) $$lnMR=\ln\left(\frac{8}{\pi^{2}}\right)-(\frac{\pi^{2}D_{eff}}{4L_{0}^{2}}t)$$

where *MR* denotes the water ratio; *D_eff_* represents the effective moisture diffusion coefficient of the material; t represents the drying time; *L*_0_ is half of the sample thickness.

#### 2.3.5. Model Analysis

Four drying kinetic models were used to fit the experimental data, and the moisture content in the drying process was analyzed. Table 1 shows the details of each mathematical model.

The fitting capability of the model was determined by the values of the RMSE, decision coefficient, R^2^, and chi-square, χ^2^. The smaller the RMSE and χ^2^ are, the closer the R^2^ is to 1. This indicates that the model has a better fitting degree. The calculation formula is as follows (6)–(8):

(6) $$RMSE={\left[\frac{1}{N}\sum_{i=1}^{N}{\left({MR}_{pre,i}-{MR}_{exp,i}\right)}^{2}\right]}^{\frac{1}{2}}$$

(7) $$R^{2}=1-\frac{\sum_{i=1}^{N}{\left({MR}_{exp,i}-{MR}_{pre,i}\right)}^{2}}{\sum_{i=1}^{N}{\left(\bar{{MR}_{exp}}-{MR}_{pre}\right)}^{2}}$$

(8) $$\chi^{2}=\frac{\sum_{i=1}^{N}{\left({MR}_{exp,i}-{MR}_{pre,i}\right)}^{2}}{N-n}$$

where *MR_exp,i_* denotes the moisture ratio obtained from the *i*th test; *MR_pre,i_* denotes the moisture ratio predicted from the *i*th test; *N* denotes the number of groups of data obtained from the test; *n* denotes the number of test model constants.

### 2.4. Determination of Color Difference

The color difference refers to the difference between the color of the dried material and the color of the fresh sample. The color of the sample was measured using a precision colorimeter (Zhengzhou North-South Instrument Co., Ltd., Zhengzhou, China). ‘L*’ denotes brightness, ‘a*’ denotes red (+) or green (−), and ‘b*’ denotes yellow (+) or blue (−). The color difference was measured by selecting the center part of each sample. To ensure the reliability of the test, all tests were repeated three times, and the average value was taken as the test value. The calculation formula is as follows:

(9) $$\Delta E=\sqrt{(L-{L^{*})}^{2}+(a-a^{*}{)}^{2}+(b-b^{*}{)}^{2}}$$

where ΔE indicates the total color difference value of the sample; L*, a*, b* indicate the brightness value, red-green value and yellow-blue value of fresh *Codonopsis pilosulae*, respectively; *L*, *a*, *b* indicates the brightness value, red-green value and yellow-blue value of dried *Codonopsis pilosulae* products, respectively.

### 2.5. Measurement of Quality Indicators

#### 2.5.1. Preparation of Extract

The preparation of the extract was changed slightly in the research methods of Jiang et al. [20]. The sample ground powder was accurately weighed after it was dried in the shade. According the ratio of material to liquid of 1: 5 (m/V), 25 mL anhydrous ethanol was added to 0.5 g of sample and placed into a 50 mL centrifuge tube. The sample was left in light-avoidant conditions on a constant-temperature shaking table to shake for 48 h (the setting parameter was 120 r/min). A freezing centrifuge was used to centrifuge the sample for 10 min at 4 °C and 4000 r/min. The supernatant liquid was collected, the volume was fixed to 25 mL with anhydrous ethanol. At 4 °C, the determination of polysaccharide content, total phenol content, total flavonoids content and antioxidation capability was carried out.

#### 2.5.2. Determination of Polysaccharide Content

A 100 μL sample extract was taken in a test tube, and the polysaccharide content was measured using the anthrone–sulfuric acid method [21]. The polysaccharide content results were recorded as the equivalent form of glucose in the slices of *Codonopsis pilosula* per unit mass. The standard curves were prepared by taking 20, 40, 60, 80 and 100 mg/L glucose solutions, respectively, and the relationship between the absorbance (y) and glucose concentration (x) was obtained as follows: y = 0.00835x − 0.163 (r^2^ = 0.99355). No sample solution was added as a blank control, and the experiment was repeated three times to ensure the accuracy of the experimental data.

#### 2.5.3. Determination of Total Phenol Content (TPC)

A sample extract of 450 μL was taken in a test tube. The total phenol content was determined using the Folin–Ciocalteu reagent method [22]. The total phenol content was expressed by the same amount of gallic acid in each quality slice of *Codonopsis pilosula*. The standard curves were prepared by taking 20, 40, 60, 80 and 100 mg/L gallic acid solutions, respectively, and the relationship between absorbance (y) and gallic acid concentration (x) was obtained: y = 0.0498x + 0.5051 (r^2^ = 0.9905). No sample solution was used as a blank control, and the experiment was repeated three times to ensure the accuracy of the experimental data.

#### 2.5.4. Determination of Total Flavonoids Content (TFC)

A sample extract of 2500 μL was taken in a test tube, and the total flavonoid content was determined using the sodium nitrite-aluminum nitrate-sodium hydroxide method [23]. The total flavonoid content results were recorded as the equivalent form of catechins in the slices of *Codonopsis pilosula* per unit mass. The standard curves were prepared by taking 20, 40, 60, 80 and 100 mg/L catechin solutions, respectively, and the relationship between the absorbance (y) and the catechin concentration (x) was obtained: y = 0.0053x + 0.037(r^2^ = 0.9984). No sample solution was used as a blank control, and the experiment was repeated three times to ensure the accuracy of the experimental data.

#### 2.5.5. Determination of Antioxidant Activity

A sample extract of 1000 μL was taken in a test tube, and the total antioxidant capacity was determined using the DPPH method [24].

### 2.6. Determination of Lobetyolin and Syringin by HPLC

#### 2.6.1. Preparation of Mixed Reference Solution

Appropriate amounts of of lobetyolin and syringin were accurately weighed, placed in a 10 mL centrifuge tube, and diluted with methanol to a concentration of 0.333 mg/mL of the control solution. A certain amount of the control solution was aspirated and produced 0.1665, 0.0666, 0.0266, 0.0106 and 0.0042 mg/mL of the mixed control solution. A standard curve was prepared, and the relationship between the peak area (y) and the concentration of the control mixture (x) was obtained as follows: y_1_ = 1520.6x1 − 1.1845 (r^2^ = 0.9995); y_2_ = 8108.7x2 + 23.547 (r^2^ = 0.9986).

#### 2.6.2. Preparation of Test Samples

Samples of approximately 0.5 g were used (through 3 screens). The samples were weighed accurately, the sample solution was placed into a 50 m long cylinder, and 25 mL of 80% methanol was added. It was then treated ultrasonically at 200 W and 60 Hz for 30 min. After cooling, the sample was weighed and rehydrated using 80% methanol. After shaking, the sample was filtered using a 0.22 μm microporous membrane to obtain a filtrate.

### 2.7. Scanning Electron Microscopy (SEM)

The specimen was prepared at dimensions of 2 × 2 mm under different drying conditions. The specimen was fixed on the SEM test bed with a conductive adhesive strip and coated using an ion sputtering instrument for 50 s.

### 2.8. Statistical Analysis

On an average basis, each group was tested three times. This study used SPSS statistics (version 22.0; IBM, Chicago, IL, USA) and Origin 8.5(Origin Lab., MA, USA). A one-way ANOVA was performed with Duncan’s test (*p* < 0.05).

## 3. Results and Discussion

### 3.1. Analysis of Drying Characteristics

#### 3.1.1. Drying Kinetics and Model

**Figure 1:**
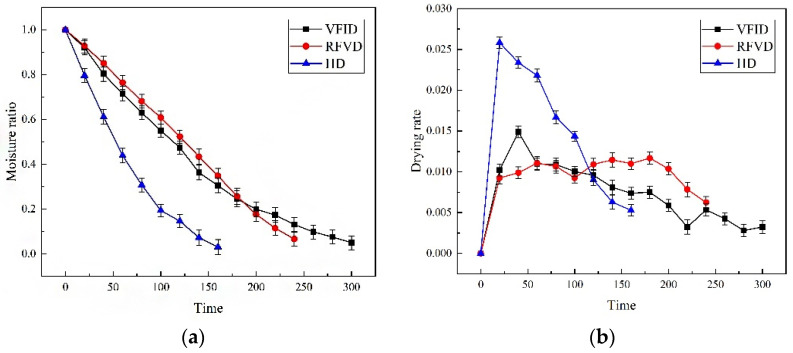
Drying curve (**a**) and drying rate curve (**b**) of *Codonopsis pilosulae* with different drying methods. VFID: vacuum far infrared drying; RFVD: radio frequency vacuum drying; HD: hot air drying. Different drying methods were carried out at a temperature of 55 °C.

The drying dynamics of *Codonopsis pilosula* under different drying modes are shown in Figure 1. The drying rate decreased with the increase in drying time, and the decreasing range was HD > VFID > RFVD, which was consistent with the drying rate. The drying time of different drying methods ranged from 300 to 1220 min (Table 2). The drying times of RMVD and VD were the lowest and highest, respectively. Microwaves can promote the vibration and friction of water molecules inside the *Codonopsis*, which can remove water faster. The vacuum reduced the vaporization temperature of the water inside the *Codonopsis*, making it easier for moisture to transfer to the surface, thereby shortening the drying time [25]. When VD was used, the drying rate of the material was low, and the heating rate was slow due to the low rate of utilization of thermal energy [26]. Compared with VFID, the time required for RFVD and HD was shortened by 20% and 46.7%, respectively.

The drying rate curve is shown in Figure 1b. The HD drying rate reached the maximum at 20 min and decreased with the extension of time. This shows that in the early stage of hot air drying, the temperature is high, the heat transfer is fast, the surface temperature of the sample rises rapidly and the humidity is carried away quickly by the hot air [27]. VFID uses radiation for heating; it can have a certain penetration capacity and can directly heat the materials [28]. During the whole drying process, the drying rate increased rapidly at 40 min, then decreased and tended to be constant at the later stage of drying. This result may be due to the rapid energy absorption and evaporation of water by the *Codonopsis* in the early stage of drying. After reaching the decreasing rate drying period, the surface structure of the material was dense due to the reduction in water content, which reduced the rate of water vapor transfer inside the material [29]. The FRVD drying process can be divided into the acceleration period, constant speed period and deceleration period. The rising speed period occurs due to the rapid rise of the internal temperature of the material in the early stage of RF vacuum drying. When the internal pressure is greater than the external environmental pressure, the dehumidification process occurs, the surface moisture evaporation is accelerated and the drying rate is increased. The constant speed period occurs because the RF heating technology has the self-balancing effect on the water content, and the surface vaporization controls the water diffusion in this process. The falling rate period occurs because the material is in the final stage of drying, and its moisture content is very small, mainly in the form of bound water. The dielectric properties of the *Codonopsis pilosula* samples decreased, and the absorbed radio frequency energy was small, making the drying rate decrease.

**Figure 2:**
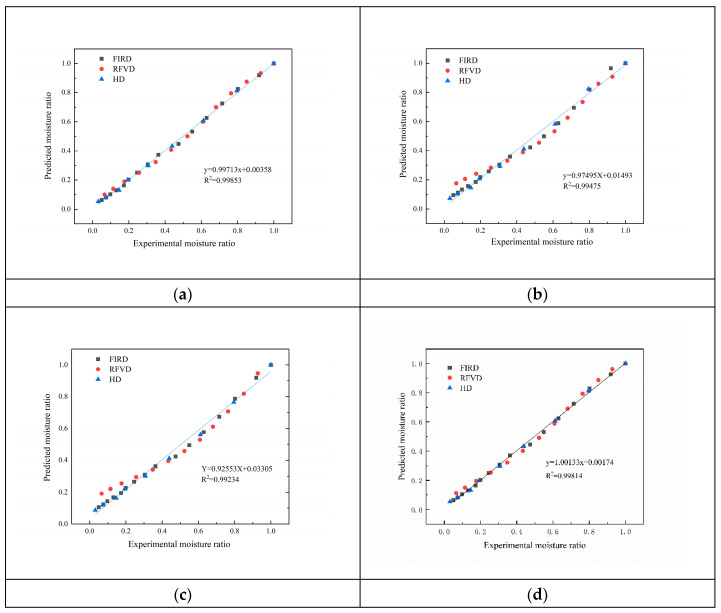
Experimental determined and predicted moisture ratio by Overhults (**a**), Verma (**b**), Dincer (**c**), and Weibull (**d**) model with different drying methods.

Table 3 summarizes the mathematical fitting results and parameter values of the four models. The coefficient of determination (R^2^), root mean square error (RMSE) and chi-square value (χ^2^) were selected as the evaluation indices for the fitting results. In the fitting model, the Weibull model has the largest R^2^ (0.99832) and the smallest χ^2^ (0.00136), followed by Verma model (R^2^ = 0.99216). Therefore, the Weibull model is the most suitable for the drying process of *Codonopsis pilosula*. For further research, the linear regression analysis of the test moisture ratio and the model-predicted moisture ratio is shown in Figure 2. The closer the slope of the regression curve is to 1 and the closer the intercept is to 0, the higher the prediction accuracy is [30]. Akin to the model fitting results, the Weibull model (d) was more accurate in predicting the experimental moisture ratio, with a slope of 1.00133 and an intercept of 0.00174. This provides further evidence for describing the drying characteristics of *Codonopsis pilosula*.

#### 3.1.2. D_eff_

It can be seen from Table 2 that the Deff is between 0.06~3.95 × 10^−8^ m^2^/s and the Deff value of RMVD is 7.05~65.83 times that of other drying techniques, indicating that *Codonopsis* had the highest mass transfer effect under this drying method. The Deff values of this study are consistent with the findings of Zogzas in which the Deff values of agricultural products were between 10^−8^ and 10^−12^ [31]. After VD treatment, the effective water partition coefficient was the smallest and the mass transfer efficiency was the worst because the material was heated at the boiling point under negative pressure. The Deff values of VFID and RFVD were not much different, indicating that the two drying methods had the same mass transfer effect during the drying process of *Codonopsis pilosula.*

### 3.2. Color Difference Analysis

The color parameter values of *Codonopsis pilosula* under different drying methods are shown in Table 2. The drying methods were different, and the ΔE of the *Codonopsis slices* is between 6.01 and 8.49. It can be seen that the color difference of the VFID samples is the largest. This is because infrared light can be quickly absorbed by molecules and converted to molecular heat. As temperature rises, Maillard’s reaction rate increases and carotenoids are oxidized and decomposed, thus becoming brighter [32]. In contrast, the L* of the samples dried using RMVD and VD was higher, indicating that these two drying methods increased the brightness value of the sample. This may be because the RMVD method takes the shortest time and reduces the Maillard reaction and the caramelization reaction of the material [33]. VD uses the internal and external pressure difference of the material to reduce the internal water boiling point of the material and reduce the thermal damage of bioactive compounds at low temperature [34]. The a* (>0) of the dried samples was between 2.71 and 4.65, and there was no significant difference in other drying methods except HD. The b* (<0) decreased compared with the ND samples except for the HD samples. From the discussion, we can see that HD-dried products demonstrated a better color-rendering performance than other drying methods.

### 3.3. Quality Analysis

#### 3.3.1. Polysaccharide

**Figure 3:**
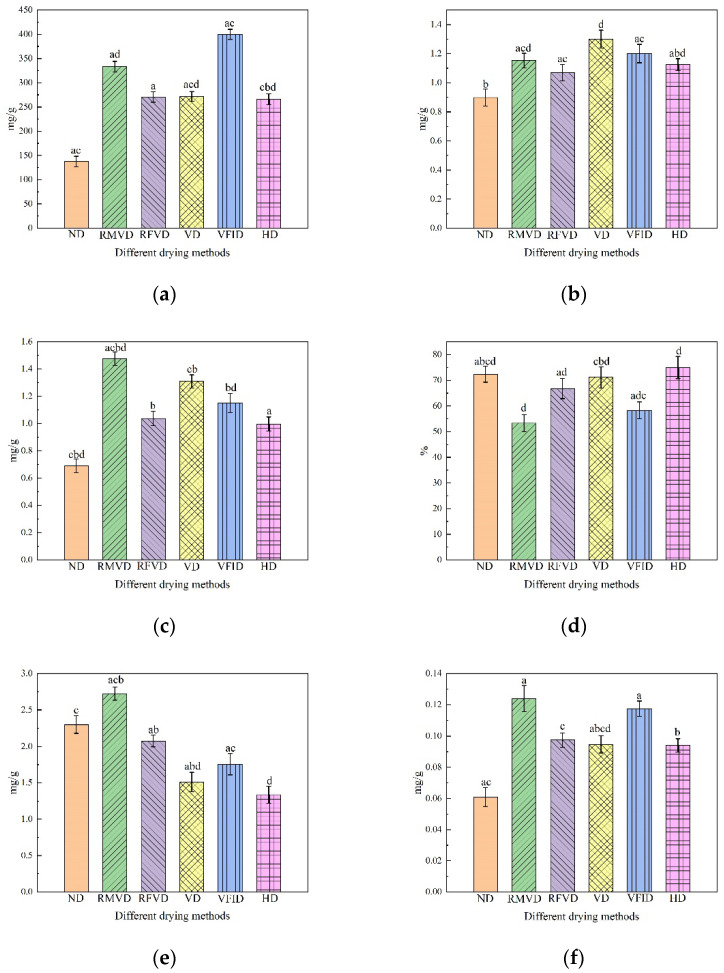
Polysaccharide content (**a**), total phenolic content (**b**), total flavonoid content (**c**), antioxidant properties (**d**), lobclyolin content (**e**), syringin (**f**) of Codonopsis pilosulae. Different letters represent significant differences (*p* < 0.05). ND: natural drying; RMVD: rotary microwave vacuum drying; RFVD: radio frequency vacuum drying; VD: vacuum drying; VFID: vacuum far infrared drying.

The effects of different drying methods on the polysaccharide content are shown in Figure 3a. It can be seen that the polysaccharide content of the ND sample was the lowest (137.47 mg/g). Different drying methods were 65.62%, 49.2%, 48.3%, 58.7% and 49.4% more than the ND samples, respectively. Obviously, the polysaccharide content of the VFID samples was the highest. This was mainly due to infrared radiation, as cellulose will be further decomposed into soluble polysaccharides and cellulose oligosaccharides. Therefore, the degradation of cellulose means that polysaccharides can be easily extracted [10]. RMVD had the fastest drying speed and the shortest drying time, and can therefore reduce the enzyme catalytic oxidation, delay the Maillard and caramelization reactions and increase the polysaccharide content [35]. There was no significant difference in the polysaccharide content among RFVD, HD and VD; that is, there was no significant difference found in the polysaccharide content.

#### 3.3.2. TPC

The effect of different drying methods on the total phenol content is shown in Figure 3b. The difference in the total phenol content was related to the heating intensity and heating time. The difference in the total phenolic content may depend on the heating intensity and duration of the heating system [36]. The total phenol content under the five drying methods was higher than that of ND (0.898 mg/g), and the retention of the VD sample was the highest (1.301 mg/g). This may be because temperature decreases the activity of oxidase and hydrolytic enzymes and avoids the loss of total phenols and the Maillard reaction causes oxidation, producing more phenolic compounds [37]. RMVD was more conducive to the retention of polyphenol content than RFVD, which is consistent with the results of Zielinska’s study, probably because microwave drying leads to an increase in the conversion rate between phenolic compounds and the detected polyphenol content [38]. The total phenol content of the VFID samples was relatively high, which may be due to the fact that the infrared radiation destroyed the internal structure of *Codonopsis pilosula* cells, reduced the permeability of cell membrane and precipitated a large number of free phenolic compounds, increasing their content.

#### 3.3.3. TFC

**Figure 4:**
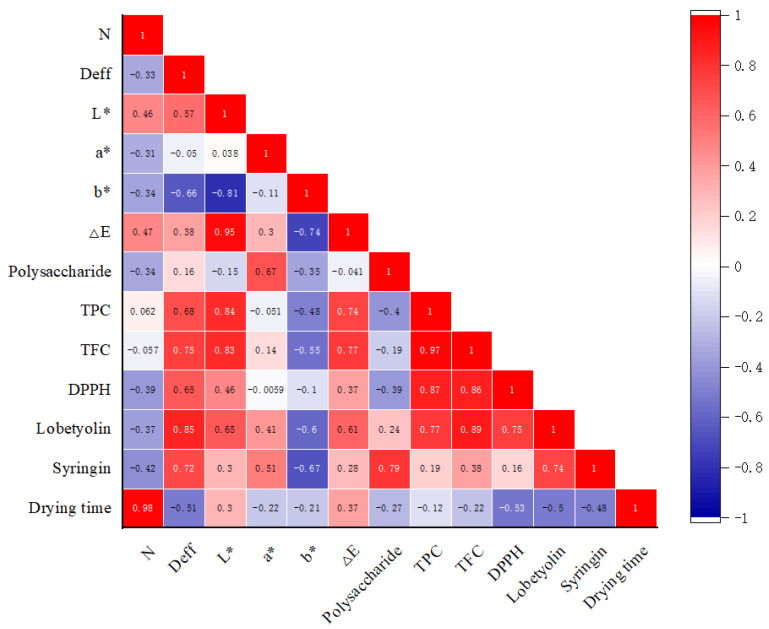
Pearson correlation analysis of *Codonopsis pilosulae* after different drying methods.

The effects of different drying methods on the total flavonoid content are shown in Figure 3c. The change in the total flavonoid content was similar to that of the total phenol content, and the content trend was RMVD (1.48 mg/g) > VD (1.31 mg/g) > VFID (1.15 mg/g) > RFVD (1.04 mg/g) > HD (1.01 mg/g) > ND (0.69 mg/g). The best retention of total flavonoids in the RMVD samples may be because the appropriate microwave radiation penetration can lead to cell rupture, making the extraction of flavonoids easier to achieve [39]. VFID samples demonstrated a relatively high content compared with RFVD and HD samples. This may be due to the far-infrared ability to break covalent bonds, which will lead to the release of a large number of antioxidants from the polymer such as flavonoids, polyphenol carotene, tannins, ascorbic acid and flavoprotein, increasing the flavonoid value in infrared dried samples [40]. According to the research, flavonoids are a major class of phenolic compounds, and increasing the content of flavonoids is beneficial to increasing the total phenolic content of the sample [41]. It can also be seen from Figure 4 that the contents of the total phenols and total flavonoids are positively correlated.

#### 3.3.4. Antioxidant Capacity

The antioxidant activity of the *Codonopsis slices* under different drying conditions was studied using the DPPH method (Figure 3d). The results showed that the HD samples had the highest DPPH scavenging ability (72.38%) under different drying treatments, which may be due to the degradation of bioactive compounds caused by mild dehydration. Additionally, the enzymatic and non-enzymatic browning products formed during the drying process may also show antioxidant properties [42]. The second-highest value was achieved by the VD samples (71.19%) because a low temperature can better preserve bioactive compounds and obtain a higher antioxidant activity [43]. Other drying treatments significantly reduced the antioxidant activity, especially in the RMVD-treated samples (53.31%). This was consistent with the results of An et al. [44]. This study showed that different thermal drying techniques reduced the antioxidant capacity of dehydrated Codonopsis slices. This decrease may be due to the effect of heating on certain components responsible for antioxidant capacity, such as phenolic compounds, flavonoids and ascorbic acid [25].

### 3.4. Active Compounds

By analyzing the contents of lobetyolin and syringin in samples that underwent different drying methods (Figure 3e,f), it was found that the contents of these two components in the RMVD samples were the highest. This may be related to the more effective drying ability and drying rate in the RMVD process, in addition to the lack of air. At a low pressure, the oxygen content of *Codonopsis pilosula* is relatively low, but high temperature, oxidation and other factors have little influence on the oxygen content. In addition, the short drying time can prevent the excessive degradation of bioactive substances in the drying process. Under different drying methods, the content of lobetyolin in the HD and VD samples was relatively low. An excessive drying time can lead to a high heat load, which may be the reason for the degradation of bioactive compounds. The VFID process increased the content of syringin (0.21 mg/g), which was 71.4% higher than that of the ND samples (0.06 mg/g). However, it was not conducive to the retention of lobetyolin (1.76 mg/g), which was 23.5% lower than that of ND (2.30 mg/g). FRVD, HD and VD demonstrated similar trends. In general, RMVD is beneficial to the retention of active ingredients, and the remaining four drying methods have a positive effect on the retention of syringin which is not conducive to the retention of lobetyolin.

### 3.5. Correlation Analysis

The correlation analysis between the parameters is helpful to explore the root cause of the mutual transformation between the appearance change in the material and the internal components. It can be seen from Figure 4 that the drying time has the greatest correlation with the unit energy consumption (r = 0.98). The Deff was positively correlated with TPC, TFC, DPPH, lobetyolin and syringin, and was significantly positively correlated with lobetyolin (r = 0.85), indicating that the better the mass transfer effect, the more conducive the process was to the retention of its content. The influence of L* on the color difference is relatively high, and b* is negatively correlated with it. The increase in the polysaccharide content was beneficial to the retention of the syringin content (r = 0.79). The contents of TPC and TFC directly affected the antioxidant activity, which was consistent with the results of Nie et al. [45]. On the whole, the correlation between the indicators further illustrates the transformation of the internal mechanism.

### 3.6. Microstructure

**Figure 5:**
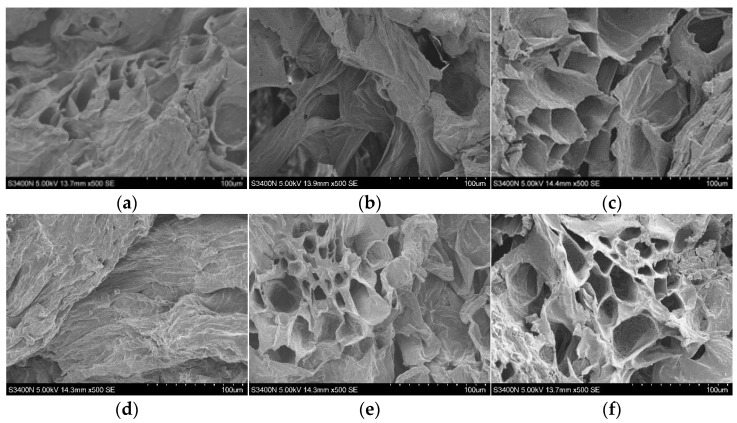
Scanning electron microscope images of Codonopsis pilosulae with different drying methods. The magnification was set as 500×. ND: natural drying; RMVD: rotary microwave vacuum drying; RFVD: radio frequency vacuum drying; VD: vacuum drying; VFID: vacuum far infrared drying. (**a**) ND; (**b**) VFID; (**c**) RFVD; (**d**) HD; (**e**) RMVD; (**f**) VD.

The microstructure changes of the *Codonopsis* samples dried using different methods were observed using scanning electron microscopy, and the results are shown in Figure 5. Compared with ND, the cell structure of the HD samples was closely arranged, and tissue shrinkage and collapse occurred. Similar findings were found in the study of cabbage by Xu et al. [24]. Due to the dielectric property of the RF material, RF can penetrate the surface of the material and generate high frequency vibrations of charged particles at a high temperature. A high heat efficiency, consistent heat transfer and mass transfer direction are conducive to the drying of a loose, porous structure formation. It was observed that the microstructure of the samples dried under RMVD and VD conditions showed irregular and large pores, which may be due to the evaporation of water by microwaves at low pressure, resulting in the expansion of the structure. This phenomenon is consistent with the results of banana microwave vacuum drying. The elimination of water during VFID resulted in a structure marked by higher cell flattening and excessive shrinkage, indicating the hardening of the slices. After long-term infrared exposure, some internal cracks were also visible. In general, different drying methods have a significant effect on the microstructure of the sample. The destruction of the internal structure of dried products and the appearance of microscopic pores can increase the yield of bioactive compounds.

## 4. Conclusions

Based on the experimental conditions, the effects of different drying methods on the drying of *Codonopsis pilosula* samples were studied. The results showed that different drying methods can significantly affect the drying characteristics and product quality. RMVD took the shortest time (18 min) to dry the sample to below the safe level of moisture content, and VD took the longest time (1220 min). The RFVD drying process can be divided into an acceleration period, constant speed period and deceleration period. Four common drying kinetic models were used to fit the drying curves of the *Codonopsis pilosula* samples dried using five drying methods, and it was found that the Weibull model could better reflect the change in the water content of *Codonopsis pilosula* under different drying methods with time. Irreversible structural changes occurred in the material during the heating process. The content of the bioactive substances in the samples was different following different drying methods. The five drying methods increased the content of polysaccharides, total phenols and total flavonoids. Compared with other drying methods, RMVD had the largest effective moisture diffusion coefficient value (3.95 × 10^−7^ m^2^/s). The contents of total flavonoids, lobetyolin and syringin under RMVD were better than those of other drying methods. The HD method had the least effect on color. The correlation analysis showed that there were different degrees of correlations among the indicators of different drying treatments of *Codonopsis pilosula*. The microscopic global view shows that VFID-dried products will show a hardening phenomenon. From the perspective of economic value and product quality, the method of drying *Codonopsis pilosula* by RMVD is worth promoting.

It is worth emphasizing that this paper studied the drying characteristics and quality characteristics of *Codonopsis pilosula* using different drying methods. We will optimize the process parameters (i.e., temperature, pressure and drying time) in the future in order to achieve the highest quality of the evaluated products.

## Figures and Tables

**Table 1:** Four common drying kinetic models.

Model	Model Equation	Parameters
Overhults	*MR* = *a·exp*[−(*kt*)*^n^*]	*a*;*k*;*n*
Verma	*MR* = *a·exp*(−*kt*) + (1 − *a*) *exp*(−*gt*)	*a*;*k*;*g*
Dincer	*MR* = *G·exp*(−*St*)	*G*;*S*
Weibull	*MR* = *exp*[−(*t/a*)*^b^*]	*a*;*b*

**Table 2:** Effects of different drying methods on drying characteristics and color parameters of *Codonopsis*.

Parameters		ND	RMVD	RFVD	VD	VFID	HD
Drying time (min)			18 ± 0.13 ^b^	240 ± 0.00 ^a^	1220 ± 0.27 ^ac^	300 ± 0.01 ^b^	160 ± 0.04 ^ab^
*D_eff_* (10^−8^ m^2^/s)			3.95 ± 0.00 ^ab^	0.29 ± 0.01 ^c^	0.06 ± 0.01 ^ac^	0.26 ± 0.05 ^abc^	0.56 ± 0.11 ^d^
Color	L*	7.21 ± 0.14 ^a^	7.60 ± 0.20 ^a^	7.75 ± 0.01 ^ab^	7.33 ± 0.03 ^b^	7.13 ± 0.28 ^ab^	5.83 ± 0.35 ^b^
	*a* *	4.62 ± 0.29 ^a^	4.65 ± 0.13 ^b^	4.24 ± 0.19 ^ab^	4.22 ± 0.20 ^b^	4.51 ± 0.23 ^a^	2.71 ± 0.14 ^ab^
	*b* *	−0.67 ± 1.14 ^ab^	−3.43 ± 0.18 ^a^	−0.88 ± 1.08 ^ab^	−2.53 ± 0.01 ^a^	−1.88 ± 0.25 ^a^	−0.55 ± 0.28 ^ab^
	△*E*	6.72 ± 0.18 ^b^	8.49 ± 0.07 ^ab^	6.98 ± 0.01 ^b^	8.46 ± 0.15 ^a^	6.10 ± 0.11 ^a^	6.01 ± 0.12 ^ab^

Note: Data are expressed as means ± standard deviation of triplicate samples. The letters in the same column reveal significant differences (*p* < 0.05) according to the Duncan test.

**Table 3:** Model fitting results.

Drying Condition	Model Name	Evaluating Indicator								
		a	b	k	n	G(g)	S	R^2^	RMSE	χ^2^
FIRD	Weibull	140.99004	1.33041					0.99807	1.9043 × 10^−4^	0.00267
RFVD		147.95568	1.63417					0.99048	9.4733 × 10^−4^	0.01042
HD		68.89244	1.27793					0.99832	1.9413 × 10^−4^	0.00136
FIRD	Dincer					1.07268	0.00775	0.98118	0.00186	0.02605
RFVD						1.0945	0.00728	0.94029	0.00594	0.06537
HD						1.04433	0.01554	0.98450	0.00179	0.01251
FIRD	Verma	1.13973		0.00826		57.5017		0.98759	0.00123	0.01595
RFVD		1.18214		0.00795		60.0018		0.95149	0.00483	0.04828
HD		1.16405		0.01729		30.88894		0.99216	9.3076 × 10^−4^	0.00542
FIRD	Overhults	0.98592		0.007	1.36254			0.99815	1.8318 × 10^−4^	0.00238
RFVD		0.95927		0.00655	1.79011			0.99226	7.7005 × 10^−4^	0.0077
HD		0.99259		0.01441	1.29117			0.99812	2.1668 × 10^−4^	0.0013

## Data Availability

The data used to support the findings of this study can be made available by the corresponding author upon request.

## Supplementary Materials

The following supporting information can be downloaded at: https://www.mdpi.com/article/10.3390/foods12061323/s1, Figure S1: Structure diagram of Rotary Microwave Vacuum Dryer; Figure S2: Structure diagram of Vacuum Far Infrared Dryer; Figure S3: The schematic diagram of Codonopsis drying and quality detection under different drying methods.
