# Supplementary material for: Effects of Different Drying Methods on the Drying Characteristics and Quality of Codonopsis pilosulae Slices
Source: Foods. 2023 Mar 20;12(6):1323. doi: 10.3390/foods12061323 (PMC10048468; doi:10.3390/foods12061323)
Supplement: Supplementary file 1 [file foods-12-01323-s001.zip › foods-2255635-supplementary.pdf]

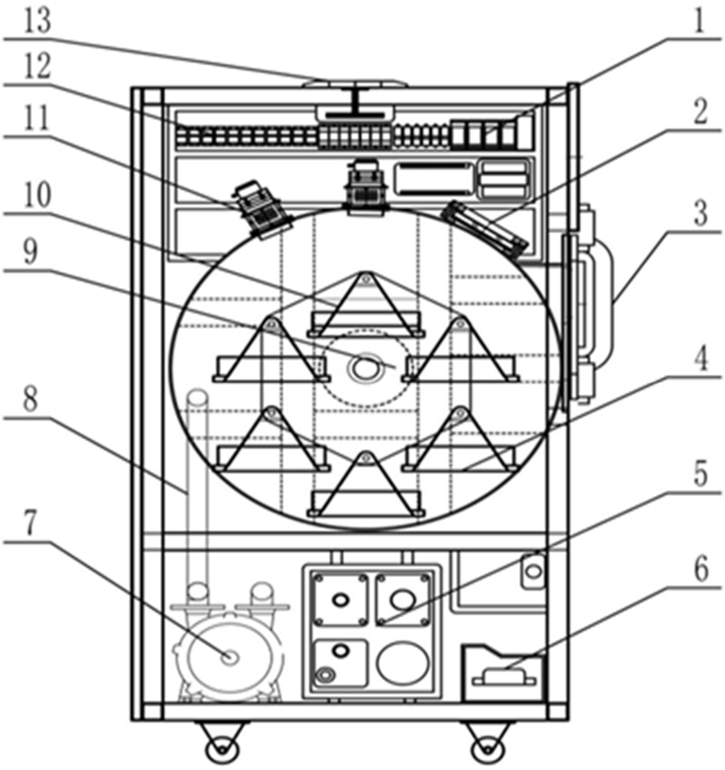

Figure S1. Structure diagram of Rotary Microwave Vacuum Dryer. 1 PLC control box; 2. Ventilation port; 3. Furnace door; 4. Dynamic material tray; 5. Vacuum pump auxiliary part; 6. Circuit protection part; 7. Vacuum pump; 8. Vacuum tube; 9. Motor;10. Material tray carrier frame;11. Microwave generator; 12. Circuit controller; 13. Louver.

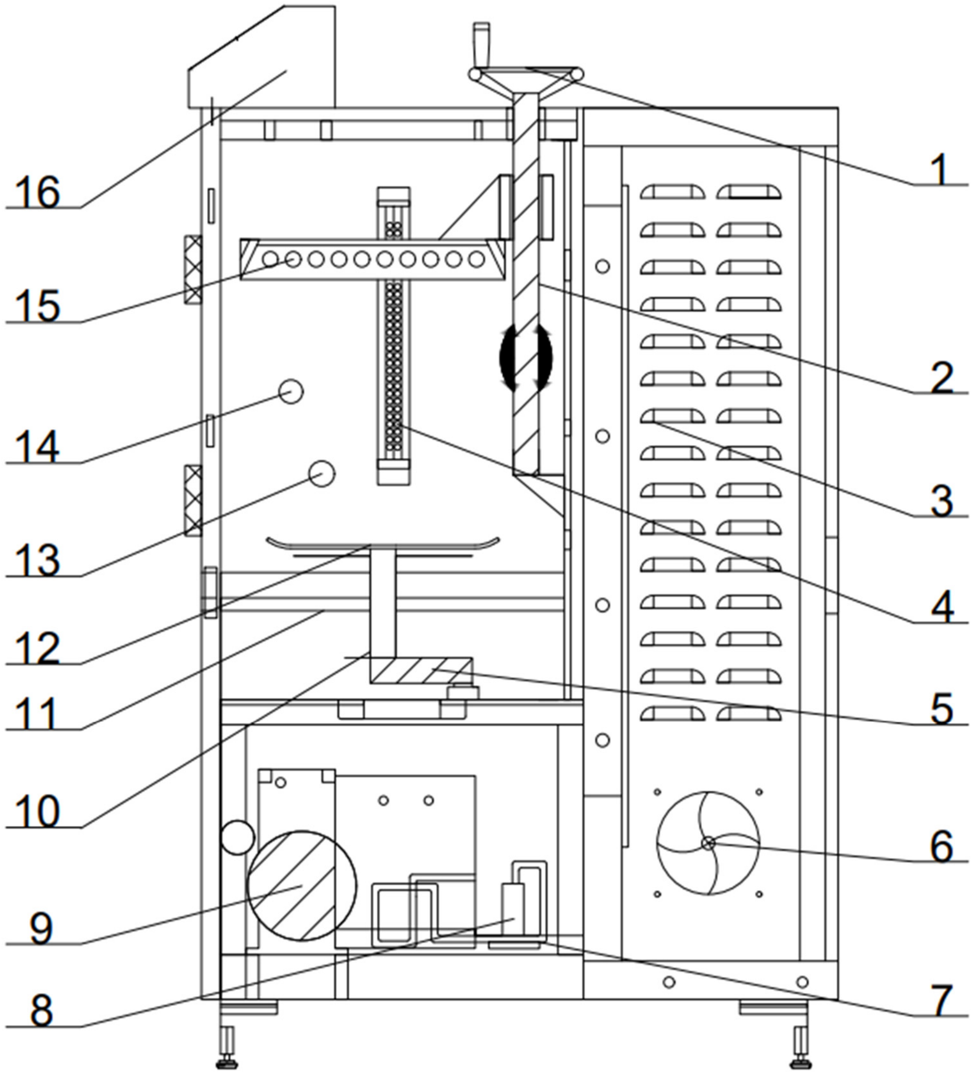

Figure S2. Structure diagram of Vacuum Far Infrared Dryer.1. Hand cranked adjustment wheel; 2. Lifting screw; 3. Electric control box;4. Chute; 5. Weighing module; 6. Fan; 7. Circulating fan; 8. Pneumatic system; 9. Vacuum pump; 10. Temperature sensor 1; 11. Thermal insulation layer; 12. Material tray; 13. Temperature sensor 2; 14. Temperature sensor3; 15. Infrared heating tube; 16.Control panel.

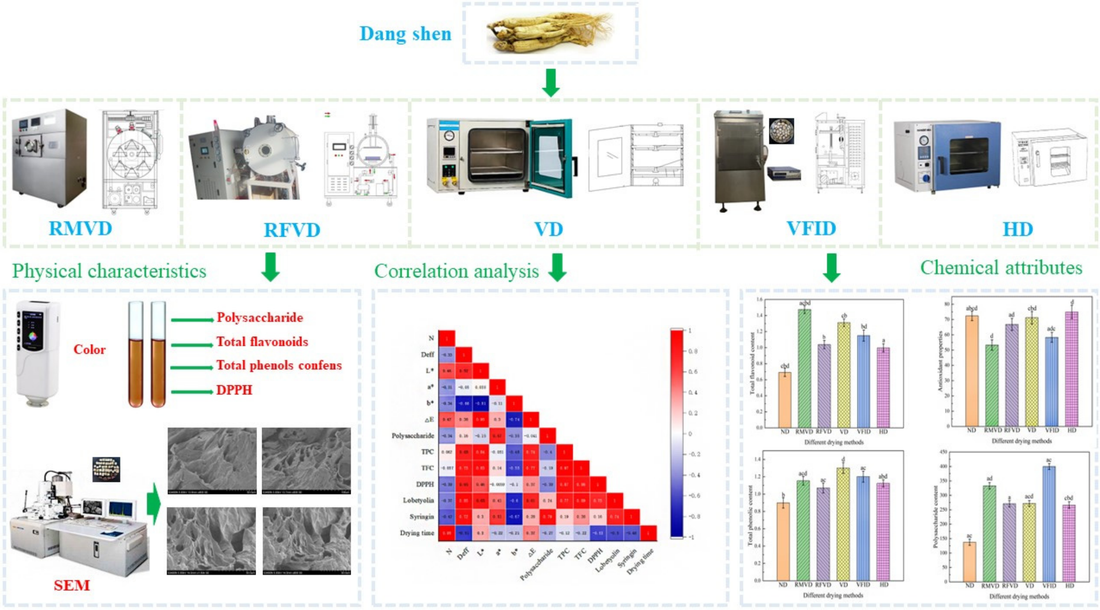

Figure S3. The schematic diagram of Codonopsis drying and quality detection under different drying methods.
